# Increases in pre-stimulus theta and alpha oscillations precede successful encoding of crossmodal associations

**DOI:** 10.1038/s41598-024-58227-z

**Published:** 2024-04-03

**Authors:** Jan Ostrowski, Michael Rose

**Affiliations:** Department of Systems Neuroscience, University Medical Center Hamburg-Eppendorf, Hamburg, Germany

**Keywords:** EEG, Theta oscillations, Alpha oscillations, Pre-stimulus interval, Crossmodal associations, Episodic memory, Phase-based connectivity, Long-term memory, Cognitive neuroscience, Human behaviour

## Abstract

A central aspect of episodic memory is the formation of associations between stimuli from different modalities. Current theoretical approaches assume a functional role of ongoing oscillatory power and phase in the theta band (3–7 Hz) for the encoding of crossmodal associations. Furthermore, ongoing activity in the theta range as well as alpha (8–12 Hz) and low beta activity (13–20 Hz) before the presentation of a stimulus is thought to modulate subsequent cognitive processing, including processes that are related to memory. In this study, we tested the hypothesis that pre-stimulus characteristics of low frequency activity are relevant for the successful formation of crossmodal memory. The experimental design that was used specifically allowed for the investigation of associative memory independent from individual item memory. Participants (*n* = 51) were required to memorize associations between audiovisual stimulus pairs and distinguish them from newly arranged ones consisting of the same single stimuli in the subsequent recognition task. Our results show significant differences in the state of pre-stimulus theta and alpha power between remembered and not remembered crossmodal associations, clearly relating increased power to successful recognition. These differences were positively correlated with memory performance, suggesting functional relevance for behavioral measures of associative memory. Further analysis revealed similar effects in the low beta frequency ranges, indicating the involvement of different pre-stimulus-related cognitive processes. Phase-based connectivity measures in the theta band did not differ between remembered and not remembered stimulus pairs. The findings support the assumed functional relevance of theta band oscillations for the formation of associative memory and demonstrate that an increase of theta as well as alpha band oscillations in the pre-stimulus period is beneficial for the establishment of crossmodal memory.

## Introduction

Patterns of ongoing brain activity can modulate how subsequent stimuli are perceived and processed^1–4^. This pre-stimulus activity has been shown to also affect how information is encoded to long-term memory, subsequently affecting memory performance itself^5–8^. Furthermore, previous research suggests that oscillatory activity across a wide range of frequency bands might be involved in the process, including low-frequency oscillations^9,10^, as well as oscillations as high as 55–70 Hz^11^. In terms of episodic memory, particularly spatial information is represented in the hippocampus through the firing of event or place cells, which is embedded in an ongoing theta rhythm of 3–7 Hz^12,13^. The information can be coded by the firing rate through the mechanism of specific receptive fields on the one hand, but also in the temporal domain by shifting the firing sequences along the phase of the overarching theta cycle (phase precession). However, these mechanisms are not only applicable to spatial information, but might also explain how associative information is encoded to long-term memory. As most of the content of episodic memory involves information integrated from different sensory modalities, it is all the more relevant that recent work extended the explanatory scope of rate and temporal coding, claiming that the same mechanism is used to form associations between discrete stimuli from different modalities and form relational networks between them, which ultimately serves episodic memory^14^. The authors suggest that event cells in the hippocampus coding for discrete events fire according to transient theta phase precession and subsequently lock onto the early theta phase. It can be assumed that the phase of theta oscillations may represent windows of short-term synaptic plasticity, and coordinate inputs from different sources. Thus, oscillatory pre-stimulus activity in the theta frequency band might be crucial for the formation of crossmodal associations in humans.

Specifically, evidence from non-invasive electroencephalography (EEG) studies suggests that increases in theta power before stimulus onset might be related to enhanced performance in tasks measuring episodic or long-term memory^10^. While some studies suggested that theta oscillations might be involved in item memory^15,16^, increased theta has also been associated specifically with better recollection of contextual information, where the association of stimuli to one of few contexts needed to be memorized^6,17^. Moreover, there is evidence suggesting that theta oscillations may play a role not only in the binding of stimuli to contexts but also in the encoding of individual associations between two stimuli^18^. In that study, participants were required to memorize individual word pairs, and were later cued by one of the words and instructed to verbalize the other half of the pair. The authors report significant increases in theta power, specifically in the pre-stimulus interval. This line of evidence gains additional support by studies utilizing intracranial EEG (iEEG), showing that the hippocampus displays increased theta oscillations associated with better memory performance^19^. However, it is still unclear whether pre-stimulus theta power is involved in modulating the encoding of individual associations between stimuli from different sensory modalities.

In this context, insights from research using animal models might provide a framework to investigate the role of theta amplitude and phase characteristics in the encoding of crossmodal associations. Supporting evidence was reported by Terada and colleagues^14^, who trained rats to perform a cue-combination task that required the integration and subsequent association of sequentially presented sound and odor stimuli. The results suggest that the firing of hippocampal neurons might represent associations between multimodal stimuli, with phase information from the theta band serving as a marker for the temporal order of discrete events. However, evidence in humans has been scarce. In one recent study, Clouter and colleagues presented participants with a multimodal memory task, in which they were required to memorize the association between a movie clip and a sound^20^. Both stimuli were presented simultaneously during encoding, but the authors manipulated the synchrony between them by fluctuating the luminance of the video and the amplitude of the sound according to a 4 Hz sine wave, while also varying their phase offset. The results show that memory performance was best when no phase offset was introduced, and that the effect was specific to the theta band. Another study followed up on these results and showed that even the single-trial phase synchrony between visual and auditory cortices, whose activity was entrained by 4 Hz fluctuations of the stimuli, could predict success in the formation of associations between different sensory information for long-term memory^21^. Thus, in addition to studies that showed a relevance of theta band amplitude modulation, the latter studies revealed an important role of phase coupling for the encoding of material from different modalities. The presented evidence further suggests that successful encoding of crossmodal associations may rely on elevated functional connectivity between the corresponding sensory areas, and that the connectivity might be centered on rhythmic components within the range of theta oscillations.

While theta oscillations seem to play a crucial role before and during the encoding of complex information to memory, there is also evidence on the involvement of oscillations in the alpha (8–12 Hz) and beta frequency bands (13–30 Hz). Alpha oscillations have been theorized to play a crucial role in selective attention^22^, which was suggested to aid encoding by inhibiting distracting information^9,23^. Investigations using iEEG measurements support this idea as increases in medial temporal pre-stimulus alpha power were reported to be associated with better memory performance^19^. Similarly, beta band activity has been observed to benefit memory encoding via inhibitory processes^24^. In the context of crossmodal associations, Scholz and colleagues reported that pre-stimulus beta oscillations in the lower bands (13–17 Hz) were indicative of successful encoding of audiovisual source memory^6^. However, it remains unclear how ongoing alpha and beta oscillations might contribute to the formation of individual crossmodal associations.

In the present study, we aimed to directly assess the relevance of pre-stimulus amplitude and phase characteristics for the formation of individual crossmodal associations between visual and auditory stimuli. In particular, pre-stimulus theta, alpha and low beta band activity, i.e. activity *before* the encoding of multimodal stimuli, might play a functional role in subsequent memory performance. We additionally examined oscillatory effects regarding post-stimulus processing as well as effects during memory retrieval. In terms of phase characteristics, phase coupling in the theta band can be hypothesized to be important during the processing of the stimuli for binding crossmodal information. Thus, if phase-based connectivity contributes to the encoding of audiovisual associative information, differences should be observed in the connectivity between visual and auditory areas. Specifically, successful memory formation would be accompanied by increased phase-based connectivity between auditory and visual areas, as compared to unsuccessful memory formation during the processing of the stimuli. Thus, we analyzed the phase-based connectivity between occipital electrodes (image-related activity)^25^ and frontocentral electrodes (sound-related activity)^26,27^.

We employed a Subsequent Memory Effects (SME) task, which is an established experimental design to investigate mechanisms related to the encoding of information to (episodic) memory. This paradigm has been used in a variety of modalities, including EEG^5,6,28,29^, magnetoencephalogram (MEG)^10,30^, and functional imaging^31,32^. The majority of the studies investigating SMEs, however, focused on the encoding of individual associations within one single sensory modality^5,17,33–35^. We modified the unimodal design to allow for individual crossmodal associations to be encoded. Participants were required to memorize associations between images and sounds while brain activity was recorded via EEG. One experimental run consisted of an encoding phase, a short distraction task, and a subsequent testing phase in the form of cued recognition. During encoding, semantically unrelated real-life images and sounds were presented simultaneously after a cue. Participants were instructed to indicate whether both individual stimuli were animal-related while making an effort to memorize the stimulus pair as a whole. After the distraction task, which required the participants to count backwards for several minutes, they were presented with the same stimulus pairs as during encoding, as well as the same number of new pairs consisting of the same individual images and sounds but randomly shuffled for new combinations. Participants needed to indicate whether they remembered the pair from the previous encoding phase or not. The stimulus pairs always consisted of individual stimuli that were presented during encoding and only the pairing was identical or different. Therefore, this task design enabled us to specifically target memory performance in terms of associations rather than individual stimuli.

## Methods

### Participants

In total, 55 healthy participants were recruited for this study. We had to exclude the data from four participants because of too many missing trials (1), low data quality (1), and hardware problems during data acquisition (2). This resulted in a final sample of *N* = 51 participants (64.71% female) that could be used for analysis, with a mean age of 24.41 years (SD = 3.82), ranging from 18 to 34 years. Participants had normal or corrected-to-normal vision and hearing ability. All participants gave their informed consent and received financial reimbursement for taking part in the study. This investigation was approved by the ethics committee of the Hamburg Medical Council (PV5893). We confirm that all experiments were performed in accordance with relevant guidelines and regulations.

### Task and procedure

**Figure 1:**
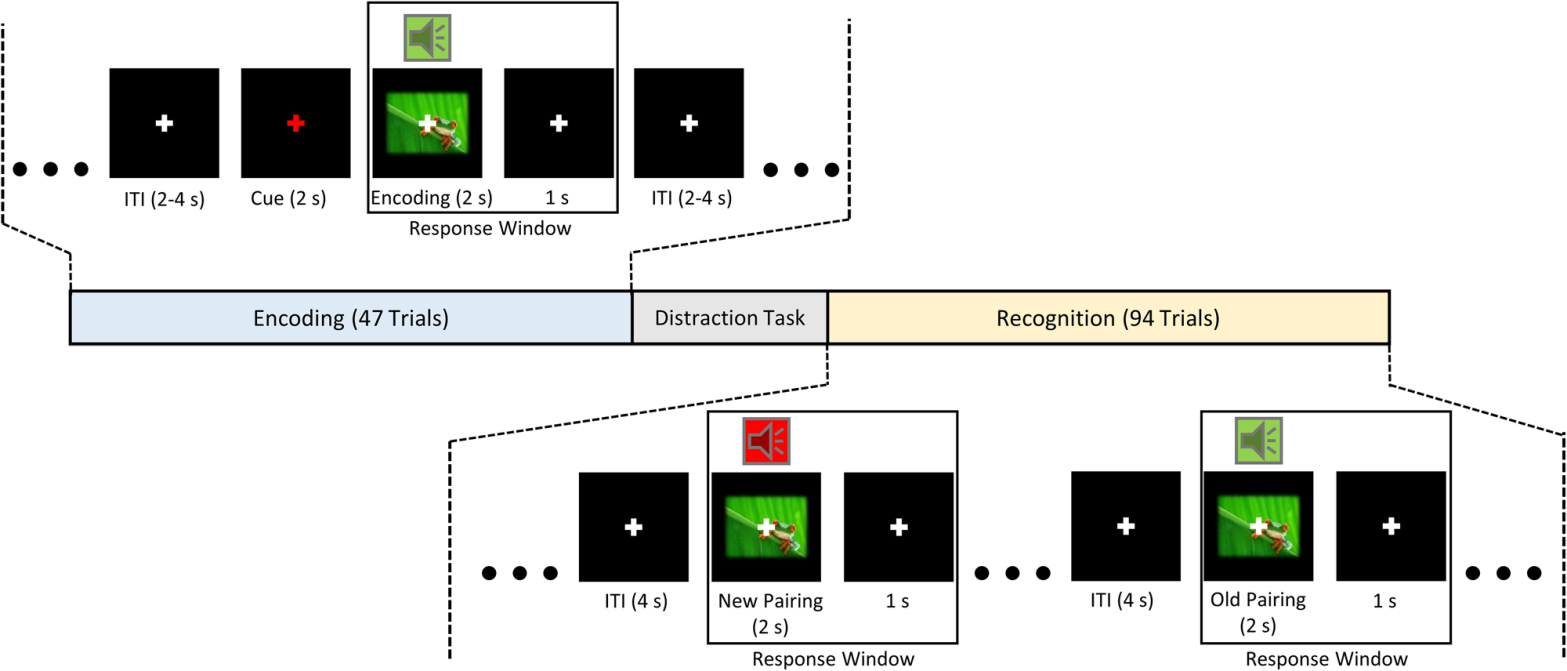
Schematic overview of one experimental run of the SME task. The run consists of an encoding phase, in which image-sound pairs needed to be memorized. This was followed by a short distraction task, where participants were required to count down in steps of 7/9/13 from 100/115/125. Subsequently, old and new pairs consisting of the same individual stimuli presented during encoding were shown, and participants indicated whether they remembered the particular pairing or not.

For this study, we implemented a Subsequent Memory Effects (SME) task consisting of three experimental runs. Each run consisted of an encoding phase, a short intermission, and a subsequent recognition phase (see Fig. 1 for a schematic overview of one experimental run). As we wanted to measure crossmodal memory, pairs consisting of one image and one sound were randomly selected from an internal stimulus database. The images were shown with a resolution of 640 × 480 pixels, a 24-bit color depth, and depicted photographs of natural or man-made scenes. A white fixation cross was layered over every image. The sounds were real-life recordings of either sounds and noises from nature (e.g. animal calls) or from a man-made environment (e.g. a honk from a car). All sounds were cropped to a duration of 2 s, and featured a bit rate of 1411 kBit/s. The pairs were pulled in a manner so that the stimulus material was unique in each run and did not repeat between runs. Specifically, each pair and individual stimulus occurred only once across the three encoding phases from the three experimental runs. Furthermore, images were paired with sounds so that congruency effects within pairs were avoided^36^. For example, while the image of a wolf could not have been paired with the sound of a wolf howl, it could have been paired with the sound of bird call or a honking car, as this would not constitute semantic congruence.

The encoding phase of each run consisted of 47 trials in which the audiovisual pairs were presented simultaneously for 2 s. The stimulus pairs were preceded by a red fixation cross with a duration of 2 s. After stimulus offset, the white fixation cross remained for a fixed duration of 1 s, which was followed by a variable inter-trial interval of 2 to 4 s. For every encoding trial, the participants were instructed to memorize the stimulus pairs, and to indicate with a button press whether both image and sound represented an animal (right mouse button) or not (left mouse button). Button presses were registered as a response during the 2 s of stimulus presentation and subsequent 1 s (Fig. 1) but were otherwise counted as a missed response. The encoding phase was followed by a brief intermission of approximately 3 min, in which the participants were asked to count down aloud from 100 (115 and 125 in the second and third run, respectively) in steps of 7 (9 and 13 in the second and third run, respectively).

In the subsequent recognition phase, the 47 audiovisual pairings from the preceding encoding phase were presented again. In addition, 47 new pairings were shown that were created from the individual images and sounds from previously learned pairs. In the instruction, participants were explicitly informed about the nature of new pairings and were further told that no new images and sounds would be introduced. All stimulus pairs were presented for 2 s, with a white fixation cross in the middle of the screen during stimulus presentation and for a fixed duration of 1 s directly after. During these 3s, association recognition was tested, as the participants were asked to indicate via button-press whether the current pair had already been presented in the preceding encoding phase (left mouse button) or not (right mouse button). The participants were encouraged to give a positive response only when confident, and to give a negative response when in doubt, to reduce the risk of false positive responses. The subsequent inter-trial interval was set to 4 s, during which a white fixation cross was visible. Across three experimental runs, participants were presented with 141 unique encoding trials and 282 recognition trials in total.

### Analysis of behavioral data

The behavioral performance of the categorization task during encoding was assessed by computing the average percentage of trials in which participants correctly identified whether both the visual and auditory stimulus represented an animal. In the recognition phase, we extracted the percentages of correctly remembered old pairings (hit), not remembered old pairings (misses), correctly rejected new pairings (correct rejections), and seemingly remembered new pairings (false alarm). As a measure of sensitivity and memory performance, *d’* was computed by calculating the difference between the z-transformed hit and false alarm rates. Then, the subject-specific *d’* values were submitted to a one-sample *t*-test to investigate the likelihood of memories of stimulus pairs being formed across the sample. Furthermore, a two-factorial repeated-measures ANOVA was used to analyze reaction times during the recognition task, with the first factor *type of pairing* (old vs. new) and the second factor *type of response* (correct vs. incorrect).

### Analysis of EEG data

#### Data acquisition

After giving informed consent and filling out a short demographic questionnaire, participants were seated in an electrically shielded and sound-attenuated chamber. We used a 60-channel electrode setup (ActiCap, BrainProducts, Gilching, Germany) to record EEG, whereas four additional electrodes were placed on the left and right temple, as well as above and below the left eye, to record vertical and horizontal EOG. The signal was referenced online to *FCz* and re-referenced offline to a common average. The ground electrode was placed on the neck below *Oz*, and electrode impedances were kept below 15 kΩ. The signal was amplified with a low cut-off frequency of 0.53 (0.3 s time constant) and recorded at a sampling rate of 500 Hz. EEG activity was recorded during all encoding and recognition phases, but not during intermissions.

#### Preprocessing and time–frequency decomposition for power analysis

The offline preprocessing of the acquired EEG data was done using the Fieldtrip toolbox^37^ for MATLAB (Release 2021a, The MathWorks Inc., Natick, Massachusetts, USA). For every trial, epochs were extracted from -1.5 s up until 2.5 s relative to stimulus onset. We used a high-pass filter at 0.5 Hz as implemented in Fieldtrip to filter out extreme low-frequency fluctuations. The data was then visually inspected and trials containing artifacts, such as high-frequency noise indicating muscular activity or spikes reminiscent of bad electrode connection, were removed. Independent Component Analysis (ICA) was used to identify components corresponding to eye blinks and lateral eye movements, which were then removed from the data. Per participant, 3.73 (SD = 1.8) components were removed on average, with most of them corresponding to muscle-related (M = 2.06, SD = 1.7) and blink artifacts (M = 1.02, SD = 0.32). Then, the data was again visually inspected, and trials that were still containing artifacts were removed. Finally, trials were separated into REM and NOTREM groups based on the responses from the corresponding recognition phase. A trial was considered as a REM trial if two conditions were met: First, the stimulus needed to be an “old” pair from the encoding phase, and the participant should have indicated that they remembered this pair. Second, the participant should have correctly identified a shuffled pair as new that contained the image shown in the original old pair. Conversely, a trial was considered a NOTREM trial if an old pair was not recognized. On average, 7.91% (SD = 7.61%) and 7.96% (SD = 7.96%) of trials were removed from the encoding data in the REM and NOTREM condition, respectively, resulting in an average of 59.29 (SD = 18.74) REM and 53.53 (SD = 18.07) NOTREM trials per participant after accounting for trials with missing responses. Bad channels were identified in the initial visual inspection, removed from the data, and interpolated using the weighted average from the neighboring channels after the ICA. Only one channel from one individual data set was interpolated in the course of the analysis.

Data from the recognition phase was preprocessed in the same manner as the encoding data. On average, 5.65 (SD = 3.14) independent components were removed from the data, the majority of which relating to blinks (M = 1.02, SD = 0.24) and muscular artifacts (M = 3.39, SD = 2.74). Here, a trial was categorized as REM if an “old” pair was presented and the participant recognized it correctly. If an “old” pair was presented and the participant did not remember it, it was categorized as a NOTREM trial. During preprocessing, 6.34% of trials with a correctly remembered stimulus pair were removed, while 6.27% of trials with not remembered stimuli were removed. This resulted in an average of 70 and 54 trials per participant, respectively.

Time–frequency decomposition was conducted in the frequency range of 1 to 40 Hz, with frequency bins of 1 Hz, and for the time interval of − 1 s to 2 s relative to stimulus onset. We chose the *mtmconvol* method for convolution as implemented in Fieldtrip^37^ with a sliding Hanning window of a fixed length of 500 ms and a step size of 100 ms. This method is a computationally more efficient version of a convolution with a complex wavelet, where the wavelet itself is constructed by convoluting the real and imaginary sine component at each frequency with the tapering function. The data and the tapered wavelet are then Fourier-transformed and element-wise multiplied in the frequency domain. At the end, the inverse Fourier transform of the result is computed. The additional 500 ms of data before and after the time interval of interest extracted during preprocessing served as padding to avoid edge artifacts from the time–frequency decomposition. For both conditions (REM, NOTREM), the resulting power values were averaged across trials for each participant. No baseline correction was applied since we were primarily interested in within-subjects differences of oscillatory power between the REM and NOTREM conditions. Furthermore, the experimental design did not allow for a suitable baseline period, as encoding-related processes could not be ruled out during the inter-trial interval. The same procedure was applied for encoding as well as recognition data.

#### Phase-based connectivity

As the theta band is thought to be involved in the process of binding incoming information^20^, we investigated whether memory performance with crossmodal stimuli could be differentiated by measures of phase-based functional connectivity. Thus, cross-spectral density data from all electrode combination pairs were extracted for the theta frequency range (3–7 Hz) in bins of 1 Hz and − 1 s to 0.5 s relative to stimulus onset in steps of 100 ms for single trials from all subjects. Next, functional connectivity was estimated using the weighted Phase Lag Index (wPLI^38^), which utilizes the imaginary part of cross-spectral densities to compute the measure and is a non-directional marker of phase-based connectivity. To avoid positive bias, we used a squared estimated of wPLI as implemented in Fieldtrip^37^.

#### Statistical analysis of EEG data

In this study, we focused on the analysis of the time–frequency EEG data acquired during the encoding phase of the experiment. Based on previous research, the main analysis focused on potential SMEs for the pre-stimulus time interval in the theta, alpha, and beta frequency band (3–30 Hz). For that purpose, we used a non-parametric permutation testing approach with cluster-based correction for multiple comparisons as implemented in Fieldtrip^37^ to statistically compare time–frequency data corresponding to REM trials to data from NOTREM trials from the encoding phase. To compare the specificity of the assumed relevance of theta band activity, the statistical analysis was calculated for the frequency spectrum of 1 to 40 Hz and a time window of − 1 to 2 s relative to stimulus onset. In this approach, paired-samples *t-*tests were conducted for every channel-time–frequency data point across participants between the REM and NOTREM condition. Adjacent data points showing significant differences between conditions (*p* < 0.05) were clustered in sets based on temporal, spatial, and spectral criteria. The sum of statistical values within each cluster was taken as cluster-level statistic**,** and the maximum of cluster-level values was chosen as the main test statistic for the comparison of conditions. Next, the Monte Carlo method was used to create a distribution of *t-*values by creating a single data set containing all trials from both conditions and randomly partitioned it into two groups. Statistical comparisons between these artificially created conditions were again conducted on the level of individual data points, and a cluster-level main statistic was computed. The drawing procedure was repeated 2000 times. On every iteration, the maximum cluster-level statistics for positive and negative clusters were extracted to create the cluster-level null-hypothesis distribution. The final *p-*value for the comparison of conditions was computed by assessing the proportion of random partitions with a larger test statistic than the one from the observed data. This procedure was repeated for all clusters found in the data, generating a *p-*value for the condition comparison for every cluster.

Building on the results from the main analysis, a correlational analysis was performed to investigate whether the magnitude of differences in oscillatory power between REM and NOTREM trials scaled with memory performance. For each channel-time–frequency data point in the range of 1 to 40 Hz and − 1 s to 2 s relative to stimulus onset, the difference in oscillatory power between REM and NOTREM trials was calculated. We then correlated the difference values with the performance measure *d’* across participants using Pearson’s correlation coefficient. To correct for multiple comparisons, the same cluster-based correction was applied to the data as described in the previous paragraph. Furthermore, we investigated the relationship between pre- and post-stimulus activity. For this purpose, we selected those data points in the theta band from the pre-stimulus (− 1 s to − 0.1 s before stimulus onset) and the post-stimulus interval (0.1 s to 2 s after stimulus onset) that showed a significant difference between the REM and NOTREM condition as suggested by the results of their statistical comparison. The same analysis was conducted for the alpha band separately. After calculating the mean difference values for the pre-stimulus and post-stimulus intervals of each participant, we used the Pearson correlation coefficient to correlate the resulting means. This process was repeated for all channels that displayed significant data points in the respective frequency bands during both pre-stimulus as well as post-stimulus intervals, resulting in correlation coefficients for each channel. The Bonferroni method was utilized to correct for multiple correlations and adjust the resulting *p*-values accordingly.

To test whether connectivity between visual and auditory areas is increased for REM trials as compared to NOTREM trials, we chose *O1, O2, Oz, PO7, PO3, POz, PO4,* and *PO8* as seed channels. For every seed channel, the connectivity data corresponding to frontocentral channels was extracted and submitted to cluster-based permutation testing, using paired-samples *t*-tests on the sample level. Frontocentral electrodes were chosen as follows: *F1, F2, Fz, FC3, FC1, FC2, FC4, C3, C1, Cz, C2, C4*.

## Results

### Behavioral results

**Figure 2:**
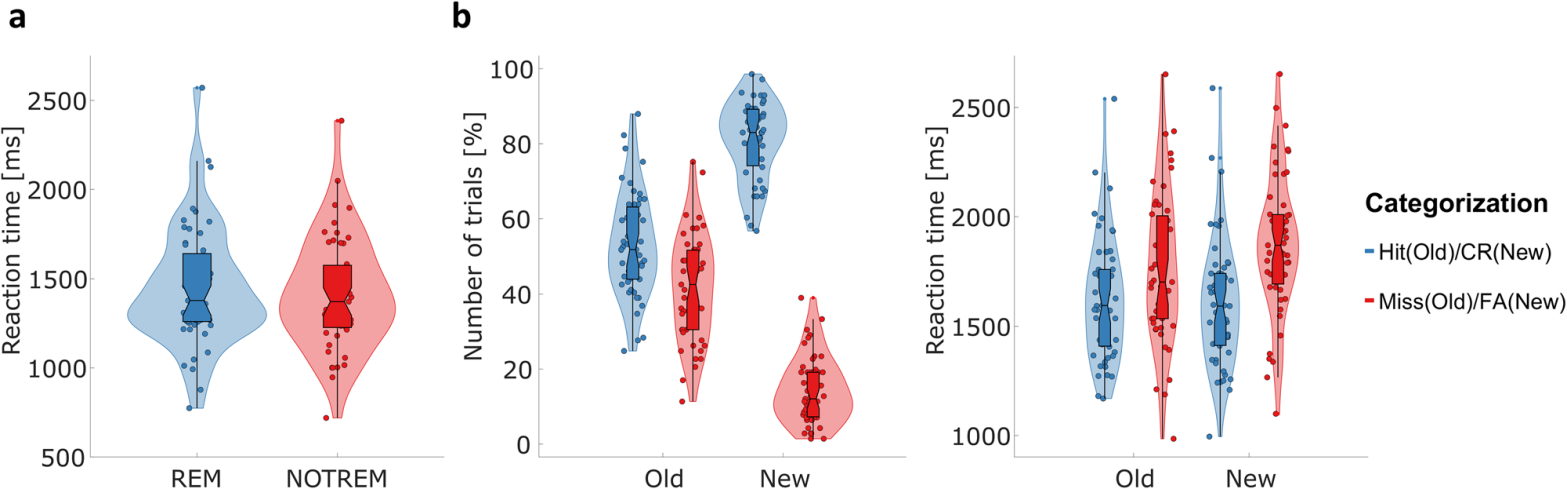
Behavioral results from the SME task. (**a**) Distribution of reaction times for the categorization task during encoding for REM and NOTREM trials across the whole experiment. (**b**) Distribution of relative number of trials (*left*) as well as reaction times (*right*) for each response category of the recognition task. For the violin plots, areas are normalized to equal within each figure. Point markers represent mean values for each participant. The horizontal line within the boxplots marks the median of the respective subset, while the notch around the median represents its 95% confidence interval. The upper and lower edge of the boxplot mark the third and first quartile of the data, respectively. The legend only refers to (**b**). *CR* correct rejection, *FA* false alarm.

In the categorization task during encoding, participants performed with an average accuracy of 92.48% (SD = 9.89%), indicating a sufficiently high compliance with the task. Reaction times from trials with later remembered and later not remembered stimulus pairs did not differ significantly, *t*(50) = 1.7711, *p* = 0.0826, although participants responded slightly faster on NOTREM trials (M = 1415.9 ms, SD = 311 ms) than on REM trials (M = 1449.9 ms, SD = 332.5 ms). See Fig. 2 for a visualization of the behavioral results.

During the recognition phase, participants had to indicate whether the presented stimulus pair had already been shown during encoding or whether it was a new pair. The hit rate was defined as the percentage of trials in which old pairs were correctly identified as known, whereas the false-positive rate was defined as the percentage of trials in which new pairs were incorrectly identified as old. On average, the participants achieved a hit rate of 52.98% (SD = 13.63%) and a false-positive rate of 14.27% (SD = 8.66%). Responses were not recorded on an average of 5.08% of trials. We calculated *d’* as a sensitivity measure for recognition performance, yielding a mean value of *d’* = 1.2456 across the sample. The results from a one-sided *t-*test revealed that the mean *d’* value was significantly different from 0, *t*(50) = 15.057, *p* < 0.001, indicating that the recognition performance was above chance across participants.

We analyzed the reaction times from the recognition phase using a repeated-measures ANOVA with the factors *type of pairing* (old vs. new) and *type of response* (REM/correct rejection vs NOTREM/false alarm). Main effects of *type of pairing*, *F*(1,50) = 10.9464, *p* < 0.01, as well as *type of response* were found, *F*(1,50) = 108.0863, *p* < 0.001. Furthermore, the interaction between these factors was also found to be significant, *F*(1,50) = 9.2168, *p* < 0.01. Thus, the correct recall of previously shown stimulus pairs and the correct identification of new stimulus pairs as new was accompanied by faster reaction times. In contrast, participants tended to respond slower in trials where old pairs were not remembered, as well as in trials where new pairs were falsely categorized as old. However, the difference in reaction times between levels of *type of response* (REM/correct rejection vs NOTREM/false alarm) was larger for new pairs (1604.4 ms vs. 1872.1 ms, *p* < 0.001) than for old ones (1621.2 ms vs. 1769.6 ms, *p* < 0.01; see Fig. 2c).

### Oscillatory results

#### Oscillatory power before and during encoding

**Figure 3:**
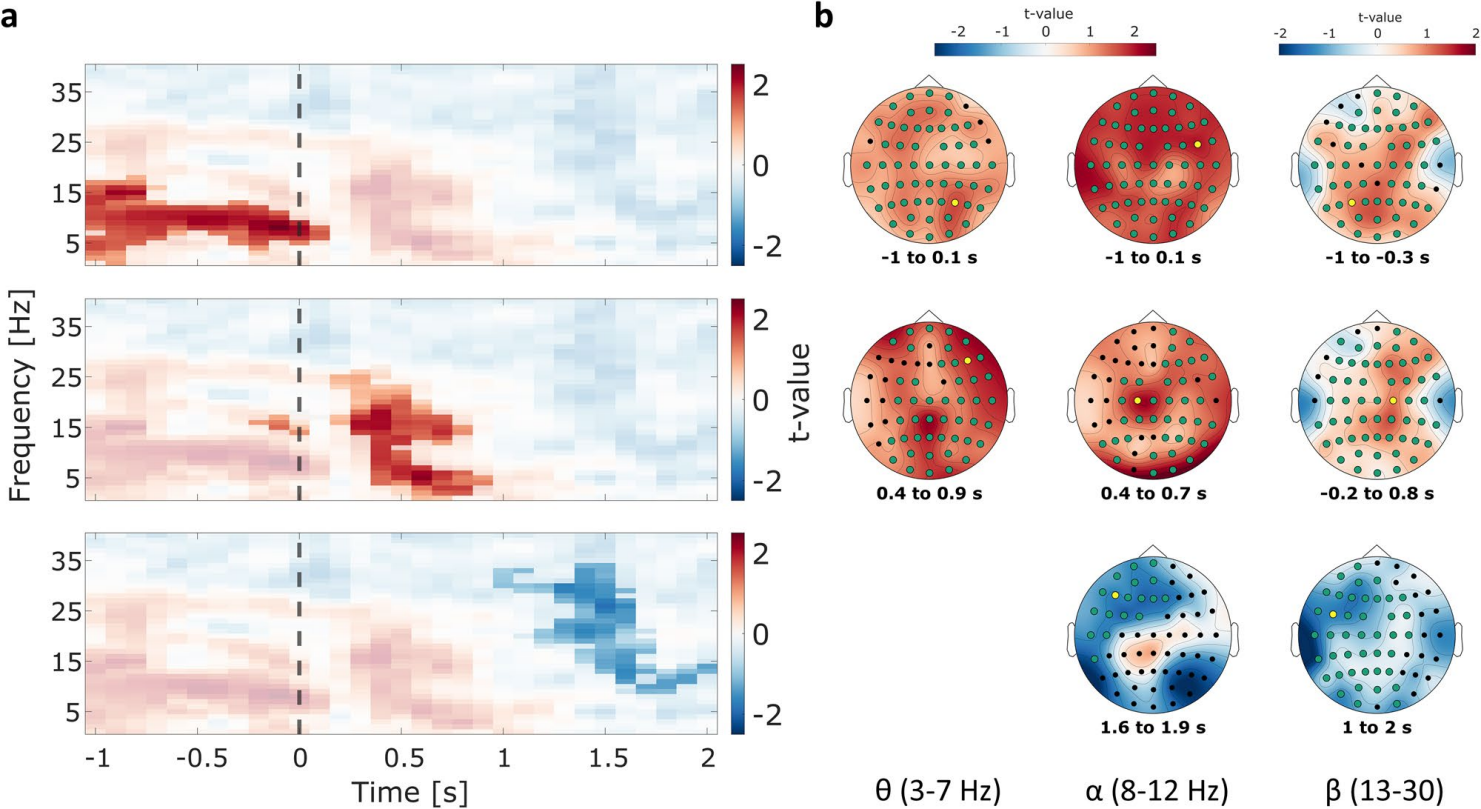
Subsequent memory effects on time–frequency power before and during encoding. Each row corresponds to one of the three distinct clusters observed in the data. (**a**) Time–frequency plots showing the results of the statistical comparison of REM – NOTREM. The vertical dashed line marks the stimulus onset. Positive *t*-values signify greater power for REM trials than NOTREM trials. Opaque data points show a significant difference at *p* < 0.5. Each plot shows one of the three distinct clusters, with *t-*values averaged over the respective electrodes that are part of the cluster: Cluster 1 (*top*) averaged over all electrodes; Cluster 2 (*middle*) averaged over all electrodes except *F7, FT7,* and *T7*; Cluster 3 (*bottom*) averaged over all electrodes except *AF4, AF8, C6, CP4, CP6, F6, F8, FC6, FT8, Fp2, Oz, P4, P6, P8, PO8, T8, TP8*. (**b**) Topographical distributions of t-values within each cluster. The columns display the distributions for the respective frequency band. Channels that are part of the respective cluster are marked in green, while yellow markers show the electrodes with maximum effect.

To assess whether oscillations before and during encoding differentiate between successful and unsuccessful memory formation, we analyzed the differences in power between REM and NOTREM trials for the corresponding time interval. The statistical comparison was conducted for the time interval of − 1 s to 2 s relative to stimulus onset, and for a frequency range of 1 Hz to 40 Hz. The analysis revealed a significant difference in oscillatory power between REM and NOTREM trials before and during the encoding of crossmodal associations (*p* < 0.05). Using a cluster-based permutation approach, a significant cluster was found in the pre-stimulus interval ranging from 1 to 18 Hz, suggesting higher oscillatory power for REM trials as compared to NOTREM trials (see Fig. 3). Similarly, increased power during REM trials was also observed during early encoding up to 0.9 s relative to stimulus onset in a frequency range of 1 to 27 Hz. Moreover, the analysis revealed an inverted effect in the late post-stimulus between 1 and 2 s after stimulus onset, spanning from 9 to 34 Hz, showing a negative cluster that did not extend into the theta band.

**Figure 4:**
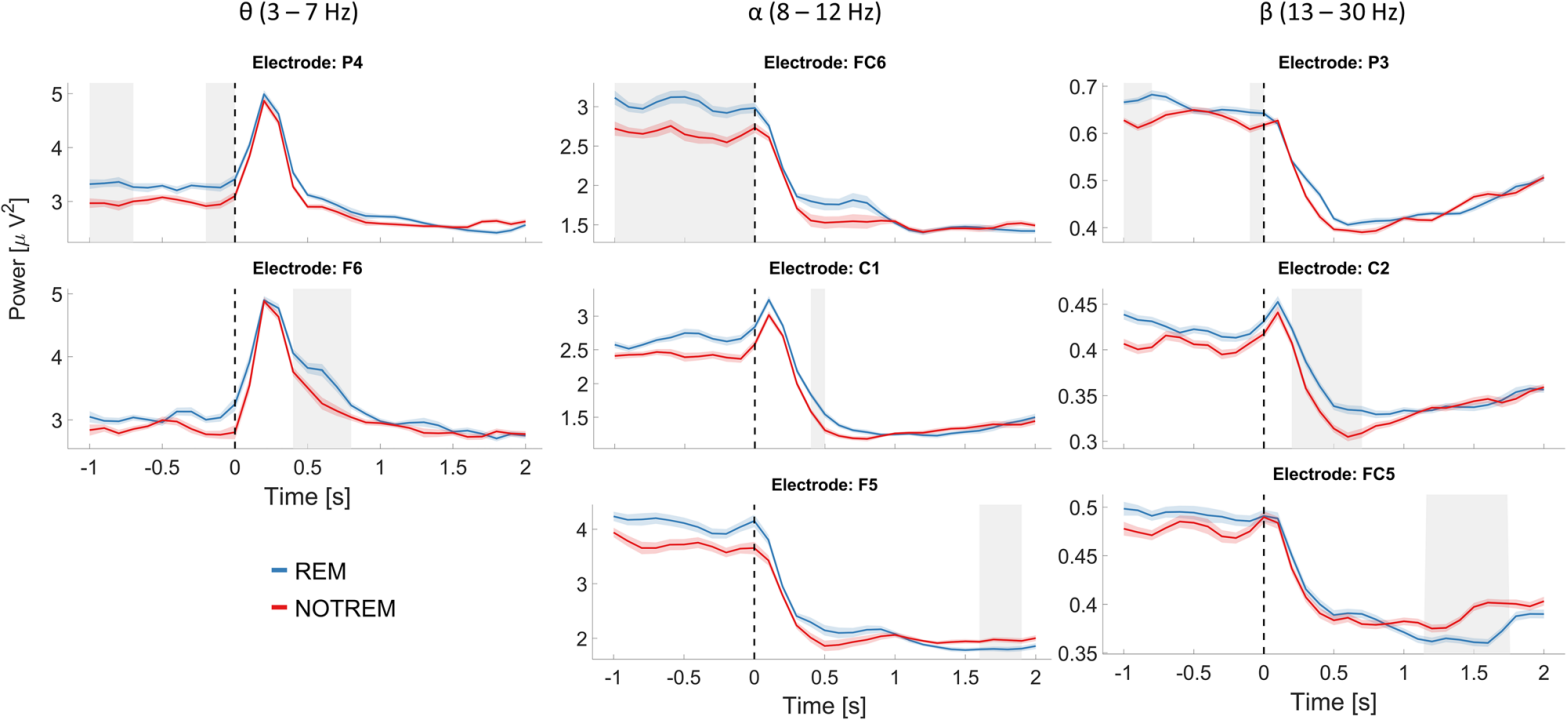
Power time courses for channels with maximum effects. The plot depicts power time courses for single electrodes averaged over the respective frequency bands. Columns denote the frequency range across which oscillatory power was averaged, whereas rows correspond to the three distinct clusters found in the statistical comparison of REM and NOTREM trials (row 1: pre-stimulus cluster; row 2: early post-stimulus cluster; row 3: late pre-stimulus cluster). The shadings around the lines mark the corrected standard error of means across participants^39^. Grey areas mark the time interval for which the difference between REM and NOTREM trials was significant in the respective cluster. The vertical dashed line marks the onset of the audiovisual stimulus pairs.

In the pre-stimulus theta range (3–7 Hz), the differences resulted to be most pronounced over the parietal as well as central areas of the right hemisphere, as well as over frontal-midline areas. The strongest effect was found at electrode *P4*, showing the highest number of data points in the theta range with a statistically significant difference between REM and NOREM trials (Fig. 4). These results indicate that theta power before stimulus onset might be beneficial for the successful encoding of crossmodal associative information. For alpha band oscillations (8–12 Hz), the effect appeared to be most pronounced right before stimulus onset within the lower frequencies of the frequency band. Here, the effects are centered on left temporal as well as right frontal cortical areas, with the maximum effect at location *FC6*. In terms of effects in the beta band (13–30 Hz), the pre-stimulus cluster incorporated only the lower frequencies between 13 and 18 Hz. In this frequency range, the largest effect was observed over left parietal and right frontal areas, most notably at electrode *P3.*

The analysis further revealed a positive cluster in the early post-stimulus interval during encoding. The differences in theta power were most pronounced in central parietal regions as well as frontal areas in the right hemisphere. The strongest effect was observed at electrode *F6* (see Fig. 4). The results indicate that higher theta power during encoding might be positively related to the formation of crossmodal associations. Similar to the pre-stimulus interval, the early post-stimulus cluster spans also the alpha as well as the beta range up to 27 Hz. The maximum effect in the alpha range for this time interval was found at electrode *C1*, while electrode *C2* showed largest effect in the beta band. In the late post-stimulus cluster, however, where REM trials displayed significantly lower oscillatory power as compared to NOTREM trials, differences in beta band activity comprised most of the cluster. Here, the effect was most notable at location *FC5*.

**Figure 5:**
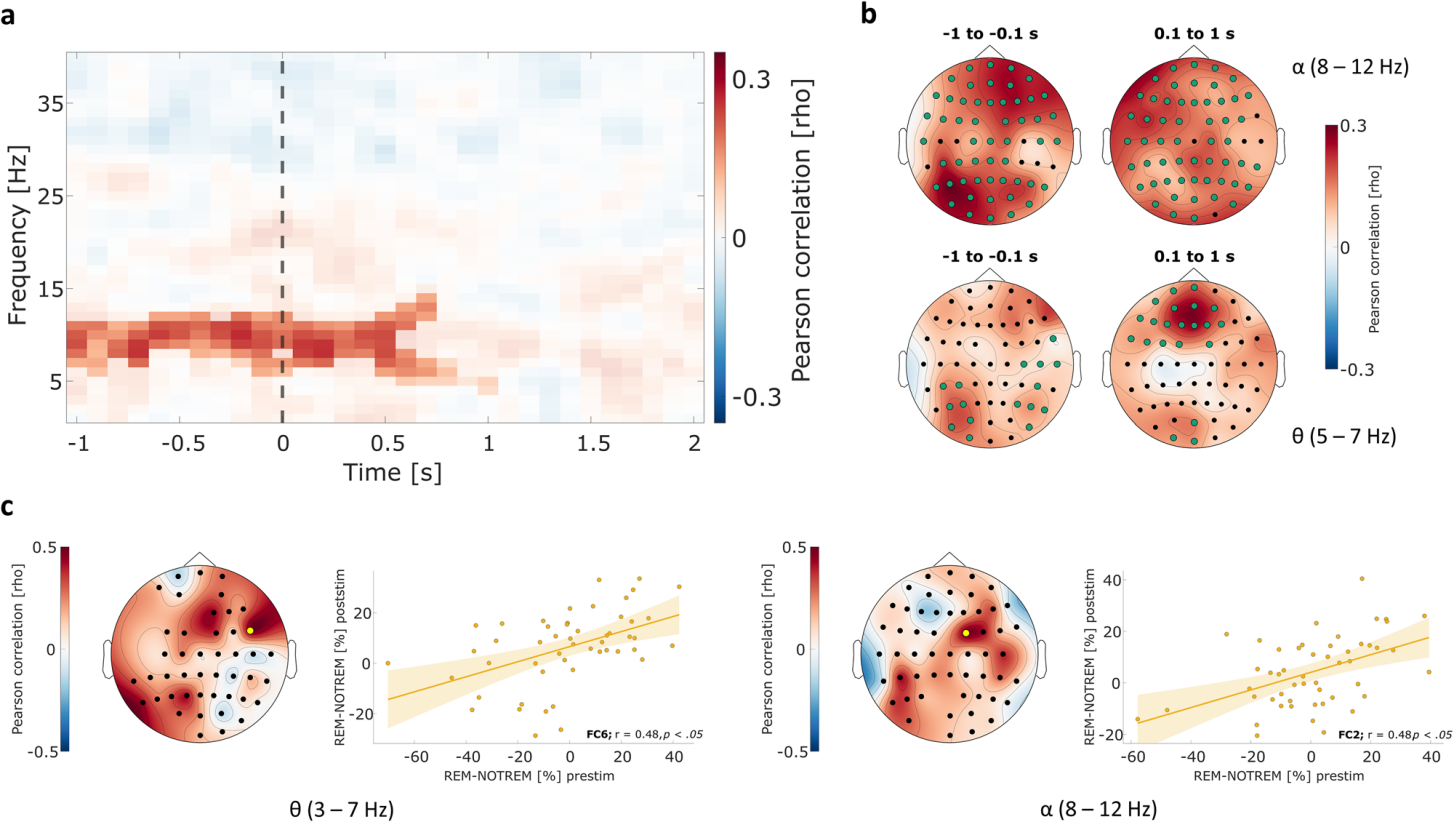
Correlation of pre-stimulus SME magnitude with memory performance and post-stimulus SME. (**a**) Time–frequency plot depicting the results of point-wise correlation of SME magnitude and memory performance measured as *d’*. Data points show individual correlation coefficients, while the opaque data points mark the significant cluster (*p* < 0.05). Positive values signify a positive correlation. The stimulus onset is marked by a vertical dashed line. (**b**) Topographical distribution of correlation coefficients averaged over the pre-stimulus (*left*) and post-stimulus interval (*right*) for the alpha (*top*) and theta band (*bottom*). Channels that are part of the cluster in this time–frequency range are marked in green. (**c**) Relationship between pre-stimulus and post-stimulus SME magnitude for theta and alpha oscillations. For each frequency band, the topographical distribution of correlation coefficients is shown (*left*). The channels with a statistically significant correlation after correcting for multiple comparisons are marked in yellow. Scatter plots show the detailed correlation for the channels with the largest effect.

In a next step, we investigated whether memory performance measured by the sensitivity index *d’* scales with the differences in oscillatory power between REM and NOTREM trials. Memory performance was correlated with the power differences for the same time–frequency range (1–40 Hz, − 1 s to 2 s relative to stimulus onset) and corrected for multiple comparisons. The analysis revealed a positive cluster spanning a frequency range of 4–15 Hz and a time interval of − 1 s to 1 s relative to stimulus onset (*p* < 0.05), indicating that greater differences between oscillatory power from REM and NOTREM trials tend to be accompanied by increased memory performance (Fig. 5a,b). For the pre-stimulus interval, the maximum correlation was observed in left parietal and right anterior frontal areas for both the alpha (8–12 Hz) and the theta band (5–7 Hz). Conversely, frontal midline areas showed the highest correlation in the theta band after stimulus onset, while for the alpha band the effect was centered around left anterior frontal and central locations. Furthermore, we were interested in the relationship between REM – NOTREM power differences before and after stimulus onset in the encoding phase for the theta band. A correlational analysis was conducted to estimate the association of power differences in the theta band between pre-stimulus and post-stimulus time intervals. After correcting for multiple comparisons, we observed a significant positive correlation at location *FC6*, *r*(49) = 0.48, *p* < 0.05 (Fig. 5c), indicating that greater pre-stimulus REM-NOTREM power differences coincided with greater post-stimulus differences. When conducting the same analysis in the alpha band, a significant correlation was found at electrode *FC2*, *r*(49) = 0.48, *p* < 0.05.

A possible confound in the analysis of pre-stimulus oscillations could arise from encoding-related activity spilling over across the inter-trial interval and influencing subsequent pre-stimulus activity. To control for that, we investigated whether the duration of the inter-trial interval could reliably predict pre-stimulus theta power on a single-trial basis by using a linear regression approach. Average pre-stimulus theta power was extracted for every REM and NOTREM trial for every participant and was used as the response variable for the model. Only data points that showed significant differences in oscillatory power between REM and NOTREM in cluster-based permutation testing were selected for averaging. Inter-trial duration was submitted as a continuous predictor, while the subsequent memory performance (REM or NOTREM) was used as a binomial predictor. The model differed significantly from a constant model, *F*(5664) = 12.4, *p* < 0.001, but the effect was exclusively driven by the predictor for memory performance, *t* =  − 4.68, *p* < 0.001. In contrast, inter-trial interval duration did not predict pre-stimulus theta-power on a single-trial basis, *t* =  − 1.62, *p* = 0.104, indicating that a confound based on oscillatory activity from preceding trials is unlikely.

#### Phase-based functional connectivity before and during encoding

Cluster-based permutation testing was used to conduct a statistical comparison of phase-based connectivity measures between REM and NOTREM trials to investigate whether visual and auditory areas display increased connectivity in REM trials as compared to NOTREM trials. Statistical estimates were obtained for every time–frequency datapoint from the combinations of seed electrodes to frontocentral electrodes. For all seed electrodes, no significant clusters were found in the data, suggesting that phase-based connectivity between frontocentral and occipital areas did not differ between REM and NOTREM trials. However, on a descriptive level, increased connectivity between occipital and frontocentral sites could be observed for trials with remembered stimuli.

#### Oscillatory power during memory retrieval

**Figure 6:**
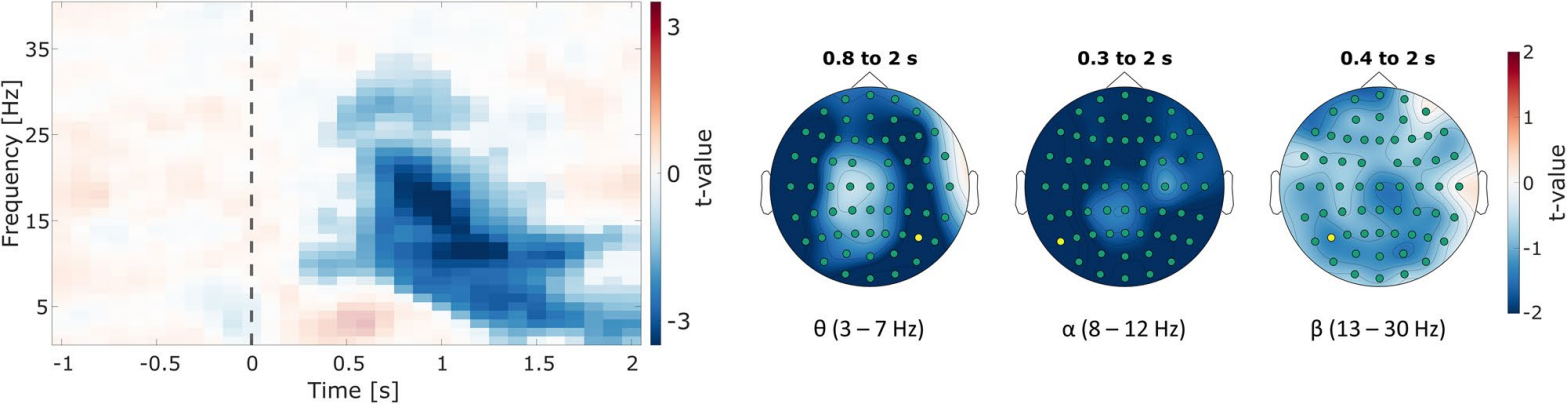
Memory effects on time–frequency power before and during recognition. Depicted are the results of statistically comparing oscillatory power between hit trials (old pairs correctly remembered) and miss trials (old pairs not remembered) from the recognition phase. The time–frequency plot (*left*) shows the *t*-values for every time–frequency data point, averaged over all electrodes. Opaque data points mark the extent of the negative cluster (*p* < 0.05). The stimulus onset is marked by the vertical dashed line. The topographies (*right*) show the topographical distribution of *t*-values averaged over the respective time intervals and frequency bands. Channels that are part of the cluster in this range are marked in green. The yellow circle marks the electrode with largest effect in the statistical comparison.

Next, we investigated whether the effects found in the time interval before encoding could also be found before memory retrieval. We used cluster-based permutation analysis to compare oscillatory power between trials where old stimuli were correctly remembered and trials where old stimuli were categorized as not known. No statistically significant difference in the theta band was found in the pre-stimulus interval. Furthermore, we report no difference in alpha or beta power for the pre-stimulus interval in that analysis. However, results show one large negative cluster in the post-stimulus interval, stretching from 0.3 s to 2 s after stimulus onset, and ranging from 1 to 34 Hz across all channels (Fig. 6). This indicates that old stimuli which were correctly remembered were associated with lower theta as well as alpha and beta power during memory retrieval as compared to old stimuli that were not remembered. The effect in the theta band is mainly driven by activity in fronto-temporal and lateral-central areas measured 1.2 s to 2 s after stimulus onset, as well as lateral parietal regions. Notably, the maximum effect was observed at electrode *P6*. For the alpha band, the effect peaked at electrode *P7*, while *P5* showed the maximum difference in the beta band. However, the onsets of the alpha and beta band effects were found to be at 0.3 s and 0.5 s after stimulus onset, respectively.

## Discussion

The present study investigated the involvement of pre-stimulus oscillatory activity and coupling in the formation of crossmodal associative memory by employing a multimodal Subsequent Memory Effects (SME) paradigm. Participants were required to memorize the association between simultaneously presented images and sounds, and the success of encoding was tested in a subsequent recognition test. We examined the differences in oscillatory power as well as phase-based functional connectivity in the pre-stimulus time interval. Notably, our analyses revealed significant differences in pre-stimulus power in the theta frequency range (3–7 Hz): trials with later remembered stimulus pairs (REM) were accompanied by increased theta as compared to trials with later not remembered stimuli (NOTREM) in the time period directly preceding the stimulus presentation. Similar differences were observed in the alpha (8–12 Hz) as well as the low beta range (13–18 Hz). Interestingly, the magnitude of SMEs in the theta and alpha band positively scaled with memory performance, suggesting a linear relationship between pre-stimulus increases in theta-alpha power and the ability to encode multimodal associations. However, our findings could not support our hypothesis regarding functional connectivity, as the modulation of phase-related connectivity in the theta band did not show a pronounced difference between remembered and not-remembered stimulus pairs. Thus, the present results point towards a memory-related function of theta band power but not phase-based connectivity. Additionally, oscillatory power before and during recognition was compared between REM and NOTREM trials, revealing decreased theta power for REM trials as compared to NOTREM trials. These effects were also found for the alpha and beta bands.

The present findings are in line with previous evidence of increases in theta power before stimulus presentation that were found to be related to more successful encoding of associative content^6,7,10,17^. Increases in theta power during encoding have been associated with better memory performance in experiments using SMEs before, especially when oscillatory data was gathered using non-invasive scalp EEG or MEG^10^. This line of evidence is supported by similar results posited by studies that did not use conventional SME contrasts but compared successful and unsuccessful memory formation based on secondary measures of episodic association, such as confidence ratings^5,40^. Interestingly, intracranial studies rarely reported exclusively positive relationships between theta power and memory performance, but instead rather both positive and negative associations^10,41,42^. In one study, however, Fell and colleagues found increases in hippocampal as well as rhinal cortex theta power in the pre-stimulus interval for later remembered words^19^. Taken together, the evidence seems to support the hypothesis of a positive association of theta power and memory performance in general, with the present results expanding the effect on audiovisual stimulus pairs and the encoding of their associations. This argument is supported by the positive relationship of SME magnitude and *d*’ as a measure of memory performance found in this study. Similar results have been presented before in healthy adults^7^, supporting the assumption that fluctuations in pre-stimulus theta power might be considered behaviorally relevant. As the maximum effect in the present study was found in right lateral parietal regions, one could speculate about the effect’s origin in multisensory processing areas. The angular gyrus, for example, has been strongly associated with the processing of multimodal information, especially combining pieces of sensory information^43^. However, this assumption should be treated with caution due to the spatial limitations of EEG measurements.

In addition to the pre-stimulus difference in theta power, a similar effect was found in the post-stimulus time interval during encoding. Theta power decreased further in NOTREM trials than in REM trials between 500 ms and 900 ms after stimulus onset. This finding is in line with previous reports on higher theta power during encoding being associated with better memory performance^44–47^. Furthermore, visual source memory was found to be accompanied by increased post-stimulus theta power^17^. The present findings show that the encoding of associations of information from different modalities can be similarly modulated by theta activity. However, judging from the topographic distributions of both pre-stimulus and post-stimulus activity, one could assume that mechanisms by which fluctuations in theta power are involved might differ. While differences in pre-stimulus theta power are centered on right parietal and anterior frontal regions, post-stimulus effects peak at central and prefrontal locations. Despite the differing topographies, REM-NOTREM differences in pre- and post-stimlus theta power were found to positively correlate. Interestingly, there was no significant correlation in right parietal locations, but in right frontal areas instead. These results indicate the possibility of differential roles of pre- and post-stimulus theta activity for the formation of crossmodal associations, while still suggesting functional connectedness. Although one cannot conclude any form of causal effect from the present results, one could speculate that pre-stimulus oscillations might affect the memorization of audiovisual associations indirectly by modifying oscillations during the encoding. Further studies are needed to investigate a potentially causal relationship and the mechanisms that might be involved.

Apart from the effects observed in the theta band, our analyses revealed similar differences between REM and NOTREM trials for alpha and low beta activity, showing higher pre-stimulus power for REM trials in both frequency bands. Consequently, the present evidence suggests that associative memory performance may also benefit from increased alpha and beta activity before the encoding of the stimulus is required. This concept aligns with previous research that has explored the links between memory and alpha or beta activity, albeit in terms of preparatory mechanisms and attention. The involvement of alpha activity, for instance, has been thought of as inhibiting task-irrelevant processes to facilitate the encoding of items^9,23,48–50^. Specifically, positive alpha SMEs in the pre- and post-stimulus intervals as observed in the present work might indicate that already encoded information is being suppressed in favor of the upcoming and then current stimulus pair, respectively^30,51,52^. As participants were presented with a multitude of audiovisual pairs that required memorization in each experimental run, it seems plausible that the encoding of a pair would benefit from the suppression of other stimuli that were shown during the encoding phase. These considerations are supported by the positive correlation of REM-NOTREM differences in alpha power and memory performance in the present work, suggesting a role of alpha oscillations and subsequently top-down processes that might be at least as relevant as the proposed theta-based mechanism of binding information.

Similarly, beta activity has been suggested to indicate cognitive preparation processes, as increased oscillatory power was measured for intentional encoding as compared to incidental^11^. These preparation processes were also proposed to be independent of the modality of the stimuli, suggesting the involvement of attentional processes^6^. It seems reasonable to assume that the successful encoding of crossmodal associations recorded in the present study would benefit from preparatory attentional processes as well, as participants were also explicitly instructed to memorize these associations. In contrast, we observed a strong negative effect in the late post-stimulus interval primarily in the beta band. Decreases in the beta band have been theorized to reflect semantic processing of to-be-encoded items^30,47^, and are assumed to originate in the left prefrontal cortex^53^. Although one must account for spatial inaccuracies when interpreting EEG results, the present findings point in a similar direction, as the maximum effect for this SME was observed in left frontal areas. One could speculate that oscillations in the beta range serve multiple purposes depending on whether or not actual stimulus material needs to be processed or not. Positive SMEs before stimulus onset might reflect stimulus-independent preparatory processes, while negative SMEs during stimulus presentation could be interpreted as a marker of semantic processing. Furthermore, as the effect is mostly centered around left frontal areas instead of visual or auditory locations, one could argue that the semantic processing takes place independent of sensory modality, but on the level of associations.

The topographies of pre-stimulus SMEs further support the notion that theta as well as alpha and beta oscillations might contribute to successful memory formation in different ways. While power differences in the theta range were most pronounced in right parietal areas, the effects in the alpha range were centered on left temporal and frontal locations. Effects in the beta range were found to be strongest around left parietal and right frontal areas. Frontal midline theta oscillations have long since been associated with episodic memory formation and retrieval^54^, while sections of the parietal lobe are usually associated with multisensory association processes^55,56^. The different topographical distributions of power differences for the pre-stimulus interval could thus be interpreted as the involvement of different cognitive processes. Considering the results from pre- and post-stimulus activity, we suggest increased pre-stimulus theta power to represent non-general preparatory processes specifically for binding, while being a marker for the actual binding of information during encoding. One could assume that these binding-specific processes are modulated by processes of task-specific inhibition in the alpha, as well as preparatory and semantic processing in the beta range. However, as evidence for spatial patterns is limited in EEG, future studies will need to test the contribution of different brain areas to the reported effects by investigating differences in BOLD signal using fMRI measurements, as well as address the question of which frequency band might be the primary driver of subsequent memory effects in the encoding of crossmodal associations.

No differences were found between REM and NOTREM trials in terms of phase-based connectivity in the pre-stimulus and post-stimulus intervals. Thus, the evidence could not support our second hypothesis, suggesting that functional connectivity between visual and auditory areas might not be beneficial for the encoding of crossmodal associations. These results are not in line with several previous studies that were able to establish a phase-based relation between auditory and visual areas by oscillating audiovisual stimulus pairs in a theta frequency for differing degrees of synchrony^20,21^. Memory performance was found to be best when stimuli were not shown at a phase offset and oscillated at a frequency of 4 Hz. Under the assumption that the phase synchrony at stimulus onset was involved in the effect that the authors found, the present results might point in the same direction, although the effect is ostensibly weaker. In another study, pre-stimulus theta connectivity within the default mode network showed the lowest prediction accuracy when predicting associative memory performance as compared to other frequency bands^33^. Interestingly, the authors reported generally higher prediction accuracies based on connectivity measures calculated for the post-stimulus interval. Notably, other lines of evidence suggest that pre-stimulus theta phase may only be connected to successful, but not to the unsuccessful encoding of associative pairings, while not observing any significant effects for pre-stimulus theta power^34^. However, the present results could also be interpreted in a way such that cortical connectivity might only play an ancillary role in binding crossmodal information for long-term memory. Instead, it could be speculated that sensory areas are phase-locked to cells in the hippocampus individually but are not functionally connected to each other for the binding process. Indeed, hippocampal projections have been suggested to drive theta oscillations in neocortical areas^57^. Furthermore, theta oscillations have been reported to reflect the dynamic integration of information from multiple sources^58^, as well as present a mechanism to functionally align the hippocampus to prefrontal cortices during recollection^40^. Additional evidence from animal studies points towards the importance of hippocampal CA1 cells for the integration of not only spatial but different kinds of sensory information from stimuli whose presentation overlapped in the time domain^14^. The authors argued that the hippocampus organizes relational networks for episodic memory, integrating phase-locked information coming in from sensory modalities. However, further research into phase-based connectivity between the hippocampus and sensory areas in the neocortex is needed, as the present results cannot account for oscillatory activity in deeper layers of the brain.

Finally, we also investigated oscillatory activity before and during retrieval. The analysis revealed that power significantly decreased in the post-stimulus interval for trials in which already-shown stimuli were remembered. This effect was found not only in the theta band but also in alpha and beta oscillations. As most studies investigating oscillatory mechanisms in episodic memory focus on effects during or before encoding, evidence on desynchronization during retrieval in the theta band has been rarely presented before. Some studies presented evidence on a positive relationship between theta power during retrieval and successful episodic memory^5^, whereas work in the context of interference and interference resolution reported positive as well as negative effects^59,60^. In one study, Pastötter and Bäuml found decreases in power that were associated with better memory performance only in high theta frequencies, while lower frequencies showed increases instead^60^. The present results are only partially in line with the previous evidence, as decreases in theta power associated with better memory performance were also found in the lower frequency of the theta band. One possible explanation could be that the power in REM trials decreased further due to longer processing time of the auditory stimuli of the pairs^61^. This decrease could then be interpreted as a positive effect for behavior. On another note, decreases in alpha power during the retrieval of associative information have been shown before. When participants were required to remember associations between words, Martín-Buro and colleagues found post-stimulus decreases between 10 and 12 Hz as early as 0.5 s after stimulus onset, predominantly in left parietal areas^62^. By comparing different degrees of successful encoding the authors suggested that decreases in alpha power during retrieval might reflect the accumulation of mnemonic evidence. Although, in the present study, successful trials were compared to trials with unsuccessful encoding, one could argue that the results might reflect a similar gradient of mnemonic evidence accumulation even for associations between different modalities, given that the peak effects were also found in left parietal areas. However, interpreting these results should be done with caution, as no hypotheses were formulated regarding the effects of oscillatory activity before and during retrieval. We recommend further research focusing explicitly on oscillatory activity during retrieval to expand understanding in that matter.

## Conclusion

This study investigated subsequent memory effects for oscillatory activity in the theta, alpha and low beta frequency range. Specifically, differences in oscillatory power and naturally occurring phase-based connectivity between later remembered and not remembered audiovisual stimulus pairs were analyzed. Importantly, theta power was found to differentiate between successful and unsuccessful encoding already prior to the stimulus presentation, i.e. in the pre-stimulus interval. The magnitude of this effect was found to be directly related to memory performance. Similar effects were observed in the alpha band and, to a lesser degree, in the beta band. In contrast, only weak evidence was observed for the assumed role of phase-based connectivity between visual and auditory brain areas for memory performance. The present findings reinforce the notion that theta band activity might be relevant in binding information from different modalities for episodic memory, and, more generally, highlight the impact of brain states before stimulus presentation on their subsequent processing. We argue that the theta-based binding mechanism might work in conjunction with inhibitory, as well as preparatory and semantic processes represented by alpha and beta oscillations, respectively, that benefit the encoding of crossmodal associations. Further research is needed to elucidate the interactions between oscillations of different frequencies, as well as the involvement of hippocampal theta oscillations in cortical processes for crossmodal associative memory.

## Data Availability

All data and code can be made available upon request through a data sharing agreement with the authors.

## References

[1] Salari N, Büchel C, Rose M (2012). Functional dissociation of ongoing oscillatory brain states. PLoS ONE.

[2] Roberts DM, Fedota JR, Buzzell GA, Parasuraman R, McDonald CG (2014). Prestimulus oscillations in the alpha band of the EEG are modulated by the difficulty of feature discrimination and predict activation of a sensory discrimination process. J. Cogn. Neurosci..

[3] Van Dijk H, Schoffelen JM, Oostenveld R, Jensen O (2008). Prestimulus oscillatory activity in the alpha band predicts visual discrimination ability. J. Neurosci..

[4] Bengson JJ, Kelley TA, Zhang X, Wang JL, Mangun GR (2014). Spontaneous neural fluctuations predict decisions to attend. J. Cogn. Neurosci..

[5] Addante RJ, Watrous AJ, Yonelinas AP, Ekstrom AD, Ranganath C (2011). Prestimulus theta activity predicts correct source memory retrieval. Proc. Natl. Acad. Sci. U. S. A..

[6] Scholz S, Schneider SL, Rose M (2017). Differential effects of ongoing EEG beta and theta power on memory formation. PLoS One.

[7] Winterling SL, Shields SM, Rose M (2019). Reduced memory-related ongoing oscillatory activity in healthy older adults. Neurobiol. Aging.

[8] Salari N, Rose M (2016). Dissociation of the functional relevance of different pre-stimulus oscillatory activity for memory formation. Neuroimage.

[9] Klimesch W, Fellinger R, Freunberger R (2011). Alpha oscillations and early stages of visual encoding. Front. Psychol..

[10] Herweg NA, Solomon EA, Kahana MJ (2020). Theta oscillations in human memory. Trends Cogn. Sci..

[11] Schneider SL, Rose M (2016). Intention to encode boosts memory-related pre-stimulus EEG beta power. Neuroimage.

[12] Buzsáki G, Moser EI (2013). Memory, navigation and theta rhythm in the hippocampal-entorhinal system. Nat. Neurosci..

[13] Huxter J, Burgess N, O’Keefe J (2003). Independent rate and temporal coding in hippocampal pyramidal cells. Nature.

[14] Terada S, Sakurai Y, Nakahara H, Fujisawa S (2017). Temporal and rate coding for discrete event sequences in the hippocampus. Neuron.

[15] Gruber MJ, Watrous AJ, Ekstrom AD, Ranganath C, Otten LJ (2013). Expected reward modulates encoding-related theta activity before an event. Neuroimage.

[16] Merkow MB, Burke JF, Stein JM, Kahana MJ (2014). Prestimulus theta in the human hippocampus predicts subsequent recognition but not recall. Hippocampus.

[17] Staudigl T, Hanslmayr S (2013). Theta oscillations at encoding mediate the context-dependent nature of human episodic memory. Curr. Biol..

[18] Haque RU, Wittig JH, Damera SR, Inati SK, Zaghloul KA (2015). Cortical low-frequency power and progressive phase synchrony precede successful memory encoding. J. Neurosci..

[19] Fell J (2011). Medial temporal theta/alpha power enhancement precedes successful memory encoding: Evidence based on intracranial EEG. J. Neurosci..

[20] Clouter A, Shapiro KL, Hanslmayr S (2017). Theta phase synchronization is the glue that binds human associative memory. Curr. Biol..

[21] Wang D, Clouter A, Chen Q, Shapiro KL, Hanslmayr S (2018). Single-trial phase entrainment of theta oscillations in sensory regions predicts human associative memory performance. J. Neurosci..

[22] Klimesch W (2012). Alpha-band oscillations, attention, and controlled access to stored information. Trends Cogn. Sci..

[23] Strunk J, Duarte A (2019). Prestimulus and poststimulus oscillatory activity predicts successful episodic encoding for both young and older adults. Neurobiol. Aging.

[24] Waldhauser GT, Johansson M, Hanslmayr S (2012). Alpha/beta oscillations indicate inhibition of interfering visual memories. J. Neurosci..

[25] Sokol S (1976). Visually evoked potentials: Theory, techniques and clinical applications. Surv. Ophthalmol..

[26] Mulert C (2007). Auditory cortex and anterior cingulate cortex sources of the early evoked gamma-band response: Relationship to task difficulty and mental effort. Neuropsychologia.

[27] Leicht G (2010). Reduced early auditory evoked gamma-band response in patients with schizophrenia. Biol. Psychiatry.

[28] Otten LJ, Quayle AH, Akram S, Ditewig TA, Rugg MD (2006). Brain activity before an event predicts later recollection. Nat. Neurosci..

[29] Gruber MJ, Otten LJ (2010). Voluntary control over prestimulus activity related to encoding. J. Neurosci..

[30] Hanslmayr S, Staudigl T, Fellner MC (2012). Oscillatory power decreases and long-term memory: The information via desynchronization hypothesis. Front. Hum. Neurosci..

[31] Park H, Rugg MD (2010). Prestimulus hippocampal activity predicts later recollection. Hippocampus.

[32] Addante RJ, de Chastelaine M, Rugg MD (2015). Pre-stimulus neural activity predicts successful encoding of inter-item associations. Neuroimage.

[33] Kim D, Jeong W, Kim JS, Chung CK (2020). Single-trial EEG connectivity of default mode network before and during encoding predicts subsequent memory outcome. Front. Syst. Neurosci..

[34] Cruzat J, Torralba M, Ruzzoli M, Fernández A, Deco G, Soto-Faraco S (2021). The phase of Theta oscillations modulates successful memory formation at encoding. Neuropsychologia.

[35] Staudigl T, Vollmar C, Noachtar S, Hanslmayr S (2015). Temporal-pattern similarity analysis reveals the beneficial and detrimental effects of context reinstatement on human memory. J. Neurosci..

[36] Parise CV, Spence C (2012). Audiovisual crossmodal correspondences and sound symbolism: A study using the implicit association test. Exp. Brain Res..

[37] Oostenveld R, Fries P, Maris E, Schoffelen J (2011). FieldTrip: Open source software for advanced analysis of MEG, EEG, and invasive electrophysiological data. Comput. Intell. Neurosci..

[38] Vinck M, Oostenveld R, Van Wingerden M, Battaglia F, Pennartz CMA (2011). An improved index of phase-synchronization for electrophysiological data in the presence of volume-conduction, noise and sample-size bias. Neuroimage.

[39] Morey RD (2008). Confidence intervals from normalized data: A correction to Cousineau (2005). Tutor. Quant. Methods Psychol..

[40] Herweg NA, Apitz T, Leicht G, Mulert C, Fuentemilla L, Bunzeck N (2016). Theta-alpha oscillations bind the hippocampus, prefrontal cortex, and striatum during recollection: Evidence from simultaneous EEG–fMRI. J. Neurosci..

[41] Greenberg JA, Burke JF, Haque R, Kahana MJ, Zaghloul KA (2015). Decreases in theta and increases in high frequency activity underlie associative memory encoding. Neuroimage.

[42] Weidemann CT (2019). Neural activity reveals interactions between episodic and semantic memory systems during retrieval. J. Exp. Psychol. Gen..

[43] Seghier ML (2013). The angular gyrus: Multiple functions and multiple subdivisions. Neuroscience.

[44] Khader PH, Jost K, Ranganath C, Rösler F (2010). Theta and alpha oscillations during working-memory maintenance predict successful long-term memory encoding. Neurosci. Lett..

[45] Hanslmayr S, Volberg G, Wimber M, Raabe M, Greenlee MW, Bäuml KHT (2011). The relationship between brain oscillations and BOLD signal during memory formation: A combined EEG-fMRI study. J. Neurosci..

[46] Osipova D, Takashima A, Oostenveld R, Fernández G, Maris E, Jensen O (2006). Theta and gamma oscillations predict encoding and retrieval of declarative memory. J. Neurosci..

[47] Hanslmayr S, Spitzer B, Bäuml KH (2009). Brain oscillations dissociate between semantic and nonsemantic encoding of episodic memories. Cereb. Cortex.

[48] Bonnefond M, Jensen O (2013). The role of gamma and alpha oscillations for blocking out distraction. Commun. Integr. Biol..

[49] Minarik T, Berger B, Sauseng P (2018). The involvement of alpha oscillations in voluntary attention directed towards encoding episodic memories. Neuroimage.

[50] Payne L, Guillory S, Sekuler R (2013). Attention-modulated alpha-band oscillations protect against intrusion of irrelevant information. J. Cogn. Neurosci..

[51] Jensen O, Gelfand J, Kounios J, Lisman JE (2002). Oscillations in the alpha band (9–12 Hz) increase with memory load during retention in a short-term memory task. Cereb. Cortex.

[52] Meeuwissen EB, Takashima A, Fernández G, Jensen O (2011). Increase in posterior alpha activity during rehearsal predicts successful long-term memory formation of word sequences. Hum. Brain Mapp..

[53] Meeuwissen EB, Takashima A, Fernández G, Jensen O (2011). Evidence for human fronto-central gamma activity during long-term memory encoding of word sequences. PLoS One.

[54] Hsieh LT, Ranganath C (2014). Frontal midline theta oscillations during working memory maintenance and episodic encoding and retrieval. Neuroimage.

[55] Sereno MI, Huang RS (2014). Multisensory maps in parietal cortex. Curr. Opin. Neurobiol..

[56] Rohe T, Noppeney U (2016). Distinct computational principles govern multisensory integration in primary sensory and association cortices. Curr. Biol..

[57] Ekstrom AD, Caplan JB, Ho E, Shattuck K, Fried I, Kahana MJ (2005). Human hippocampal theta activity during virtual navigation. Hippocampus.

[58] Feng T, Silva D, Foster DJ, Snyder SH (2015). Dissociation between the experience-dependent development of hippocampal theta sequences and single-trial phase precession. J. Neurosci..

[59] Khader PH, Rösler F (2011). EEG power changes reflect distinct mechanisms during long-term memory retrieval. Psychophysiology.

[60] Pastötter B, Bäuml K-HT (2014). Distinct slow and fast cortical theta dynamics in episodic memory retrieval. Neuroimage.

[61] Robinson CW, Chadwick KR, Parker JL, Sinnett S (2020). Listen to your heart: Examining modality dominance using cross-modal oddball tasks. Front. Psychol..

[62] Martín-Buro MC, Wimber M, Henson RN, Staresina BP (2020). Alpha rhythms reveal when and where item and associative memories are retrieved. J. Neurosci..

